# Converting a Periplasmic Binding Protein into a Synthetic Biosensing Switch through Domain Insertion

**DOI:** 10.1155/2019/4798793

**Published:** 2019-01-03

**Authors:** Lucas F. Ribeiro, Vanesa Amarelle, Liliane F. C. Ribeiro, María-Eugenia Guazzaroni

**Affiliations:** ^1^Department of Biology, Faculdade de Filosofia, Ciências e Letras de Ribeirão Preto, University of São Paulo, São Paulo, SP, Brazil; ^2^Department of Microbial Biochemistry and Genomics, Biological Research Institute Clemente Estable, Montevideo, Uruguay; ^3^Department of Biochemistry and Immunology, Faculdade de Medicina de Ribeirão Preto, University of São Paulo, São Paulo, SP, Brazil

## Abstract

All biosensing platforms rest on two pillars: specific biochemical recognition of a particular analyte and transduction of that recognition into a readily detectable signal. Most existing biosensing technologies utilize proteins that passively bind to their analytes and therefore require wasteful washing steps, specialized reagents, and expensive instruments for detection. To overcome these limitations, protein engineering strategies have been applied to develop new classes of protein-based sensor/actuators, known as protein switches, responding to small molecules. Protein switches change their active state (output) in response to a binding event or physical signal (input) and therefore show a tremendous potential to work as a biosensor. Synthetic protein switches can be created by the fusion between two genes, one coding for a sensor protein (input domain) and the other coding for an actuator protein (output domain) by domain insertion. The binding of a signal molecule to the engineered protein will switch the protein function from an “off” to an “on” state (or vice versa) as desired. The molecular switch could, for example, sense the presence of a metabolite, pollutant, or a biomarker and trigger a cellular response. The potential sensing and response capabilities are enormous; however, the recognition repertoire of natural switches is limited. Thereby, bioengineers have been struggling to expand the toolkit of molecular switches recognition repertoire utilizing periplasmic binding proteins (PBPs) as protein-sensing components. PBPs are a superfamily of bacterial proteins that provide interesting features to engineer biosensors, for instance, immense ligand-binding diversity and high affinity, and undergo large conformational changes in response to ligand binding. The development of these protein switches has yielded insights into the design of protein-based biosensors, particularly in the area of allosteric domain fusions. Here, recent protein engineering approaches for expanding the versatility of protein switches are reviewed, with an emphasis on studies that used PBPs to generate novel switches through protein domain insertion.

## 1. Introduction

A biosensor consists essentially of an input module, responsible for interacting with the target molecule, and an output module which transforms the molecule recognition into a detectable signal [1]. Over the past decade, the interest in developing biosensors capable of sensing and responding to small molecules has shown tremendous progress, fuelled by the desire to detect disease biomarkers, pathogens, and environmental toxins, to measure metabolite concentration, to create efficient high throughput screening methods, and also to generate therapeutic response triggered by a specific small molecule [2, 3]. Despite the great biotechnological potential, there is no general strategy for the construction of biosensors. Many of the current methods use a limited repertoire of naturally occurring ligand-binding proteins to couple the binding of the target molecule to the output signal, restricting the scope of target molecules that can be detected [4].

Proteins possess properties that make them ideal recognition modules, such as impressive specificity, affinity, and versatility. In protein biosensor development, two general approaches can be highlighted to convert a binding event into a detectable signal. (i) The first approach encompasses the immobilization of the protein on a piezoelectric, optical, electrochemical, or electrochemiluminescence device. In this case, the binding events are recorded by the difference in a physicochemical change. This approach is known as a two-component system, and it has the ability to detect molecules that cannot be imported into the cytoplasm. Nonetheless their use as biosensors is limited by the risk of cross-talk, surface adsorption, and the requirement of extra detection equipment [5]. (ii) The second approach involves a single protein that can be used as both recognition and transduction module. Compared to the two-component systems, this arrangement of sensor and effector in one molecule is simpler and more effective and it reduces potential issues associated with surface adsorption and the dependency of the complex and expensive detection equipment [6]. This strategy is ideal for whole-cell biosensor applications [7]. Whole-cell biosensors can provide the advantages of rapid and sensitive analysis for* in situ* monitoring with cells [8–10]. Single-protein biosensors can be expanded through the engineering of proteins in which the molecular recognition is coupled with a detectable protein function.

**Figure 1:**
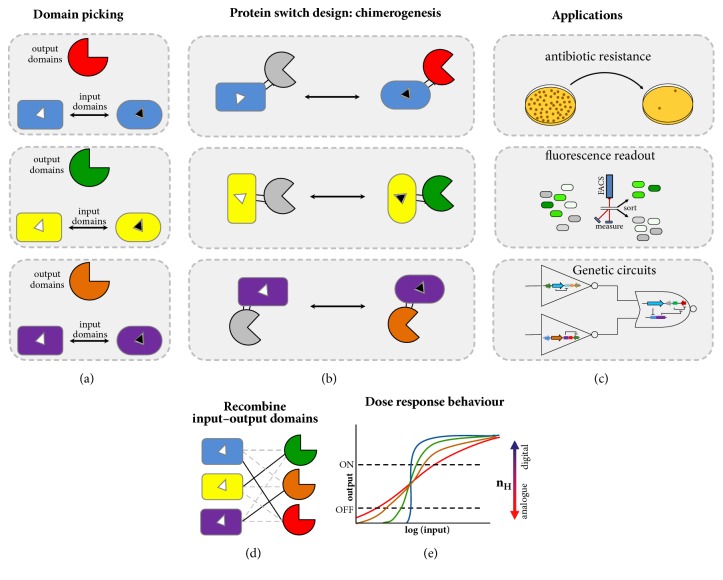
Schematic depiction of the creation of protein switches by domain insertion. (a) The input or output domains are selected according to the desired application and also structural characteristics. Generally, the protein to be inserted has proximal N- and C-termini. Ligand-mediated conformational changes in the input domain may allow molecular communication between fused domains through conformational coupling. Different colors represent different domains. (b) After chimerogenesis, by domain insertion, the protein switch has the two domains fused in such a way that the activity of the output domain is regulated by the input domain's recognition of an input signal. (c) Depending on the coupled protein functions, the switches can be used as powerful tools for several applications, such as diagnostics, high throughput screenings, and integrating genetic circuits. The grey color of the output domain indicates that the protein is inactive. The signal that modulates the switch is showed as a black triangle. (d) A significant question in the design of novel protein switches is finding the correct combination between the input/output domains which allows the signal/response coupling.** (**e) According to the molecular switch sensitivity, the protein can show a digital- or analogue-like behavior. Hill coefficients (n_H_) > 1 show a cooperative response.

A promising approach to design new generation single-protein biosensors is to expand the toolkit of the allosteric molecules known as “protein switches.” A typical protein switch is a biomolecule that can change between two or more distinct conformations (or conformational ensembles) in response to a specific stimulus [11, 12]. These changes modulate their active state – output − (e.g., enzyme activity, ligand affinity, fluorescence, and oligomeric state) in response to a binding event or physical signal – input – (e.g., small molecule, pH, covalent modification, and light). A usual approach to design switchable proteins has been to fuse a protein able to recognize an input signal (e.g., periplasmic binding proteins) with a protein whose function one desires to create an input-dependent response (Figures 1(a)-1(c)). Since protein switches can transduce an input signal into a functional response, they are logical targets to build biosensors.

Biological systems can be described as an interacting network of molecules organized in complex circuits. Protein switches are key components able to couple cellular functions. Their behavior is similar to the natural allosteric proteins, exhibiting remarkable attributes that make them an extraordinary model to design biosensors, such as high specificity and affinity, reversible signal transduction, versatility, and fast response, acting in millisecond to microsecond timescale [13, 14] which is faster than inducible gene expression-based systems (seconds to hours) [15]. In addition, unlike a single domain with linear response, allosteric switches can produce a cooperative connection of the input, leading to a finer adjustment of the output. Thus, components with highly sensitive switch behavior resemble a digital response, providing an input detection threshold in which small changes in the input concentration lead to large changes in the output response (Figure 1(e)). All these attributes are important to continuous and real-time molecule detection even inside living cells.

Synthetic proteins switches are engineered to show a user-defined input and output recognition/response. Engineered protein switches have been used in a wide variety of applications such as biosensors [6, 16–19], cancer or diabetes therapeutics [20, 21], biomass degradation [22, 23], recognition of cell signaling elements [24–26], and control of gene expression and genome editing [27–30]. However, the main challenge to design a protein switch is overcoming the problem of how to couple input and output functions, both physically and functionally, so that binding of the analyte produces a detectable signal. The science to engineer protein switches by coupling any desired input to output domains would enable the rewiring of cellular circuitry according to bioengineer's goals.

Despite the wide potential, protein switches have not been extensively explored because of the scarcity of universal engineering strategies and the difficulty to design a protein that responds to a signal unrelated to its function, becoming more (or less) active in the presence of this signal. In this review we will discuss recent studies that used protein engineering approaches, domain insertion and directed evolution, to recombine nonhomologous proteins generating molecular switches. We will be emphasizing those studies that expanded the toolkit for allosteric switches using the superfamily of proteins known as periplasmic binding proteins (PBPs), whose members are able to bind to diverse ligands. A summary of the engineered switches, along with their properties, is provided in Table 1.

## 2. Engineering Switches by Protein Domain Insertion

Protein domains are evolutionarily conserved polypeptide units that usually present independent functional or structural properties. More than two-thirds of the proteins found in prokaryotes and eukaryotes contain multiple domains [31]. Moreover, protein domains can act as structural reservoirs to generate new protein architectures [32] and can be used as building blocks to design new proteins with expanded biotechnological applications [11, 12, 22, 23, 33–37]. Multidomain proteins can be engineered by domain insertion in such a way that a domain (insert) is spliced into another domain (acceptor) either at a specific position or by random insertion. Structural coupling among the combined domains can emerge from the recombination of insert and acceptor domains, with the emergence of new functions [38]. The first example of successful insertion of one protein into another was published in 1990, when Ehrmann and colleagues inserted the alkaline phosphatase from* Escherichia coli* into the membrane protein MalF also from* E. coli*. This engineered chimera was constructed as a tool for examining membrane protein topology [39]. Furthermore, different from end-to-end fusion in which domains are linked by a single contact point, when two domains are fused by domain insertion, they are linked by peptidyl bonds at two contact points with more intimate connection between the two proteins. This double contact can increase the protein stability of generated chimeras [23, 35, 40, 41], and the intimate connection can be used to couple the functions of two fused proteins.

Proteins switches can be designed to emulate the behavior of natural allosteric proteins, but with user-defined input and output signals. Allosteric protein switches can be created by fusing two domains in such a way that the activity of the output domain is regulated by the input domain's recognition of an input signal [12, 34] (Figures 1(a)-1(c)). A significant challenge for designing novel switches is finding the correct match between the input/output domains which allows the signal/response coupling (Figure 1(d)).

For the creation of protein switches by domain insertion, the acceptor protein has to be a discontinuous protein, its linear sequence can be interrupted by anotherinserted protein, which is fairly prevalent in nature [34, 42]. In addition, the N- and C-terminals of the inserted protein should be close enough to permit the insertion without disrupting the protein structure [34]. This condition is not as restrictive since at least 50% of all single domain proteins have their N- and C-termini proximal (<10 Å) [11, 34, 43]. However, the exact fusion to create a switch is difficult to predict. Thus, a directed evolution approach is preferred in which an insert gene is randomly inserted into an acceptor gene, to create a library of gene fusions encoding fused proteins. These fused proteins are then subjected to selections or screens to find chimeric proteins that possess signal-dependent response [11].

**Figure 2:**
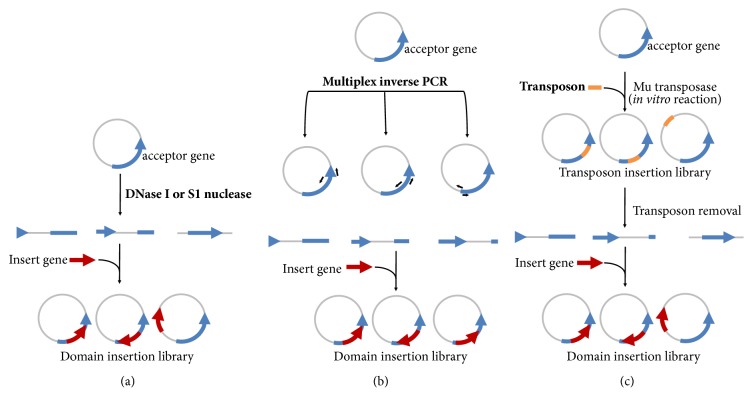
Schematic representation of the strategies used to generate random domain insertion libraries. (a) DNase I or S1 nuclease, in specific conditions, can generate a single break at the plasmid containing the acceptor gene. (b) Multiplex inverse PCR can open up the plasmid at targeted positions in the acceptor gene. (c)* In vitro* transposition uses an engineered transposon to randomly linearize the plasmid. The gene coding for the insert domain is ligated in all the approaches.

Random insertion libraries are usually created by DNase I or S1 nuclease [44], multiplex inverse PCR (MIP) [11], or* in vitro* transposition [45] (Figure 2). Nuclease-digestion generates a single double-stranded break throughout the plasmid that encodes the acceptor gene, while MIP uses designed primers to “open up” the plasmid to facilitate domain insertion. MIP can be used for random domain insertion (i.e., inverse PCR at each codon) or for rational design (i.e., achieved by inverse PCR only at designated positions). In* in vitro* transposition an engineered transposon is randomly inserted into the plasmid carrying the acceptor gene by an* in vitro* transposase reaction. Then, the transposon is removed by restriction digestion and single break plasmids are size selected. Both nuclease-digestion and* in vitro* transposition methods can generate insertions outside of the coding sequence, deletions, and a significant fraction of the library harboring out-of-frame and undesired fusions, even though small in-frame deletions contribute to increasing library diversity. Therefore, both strategies are best combined with a robust screening system. The inserted gene, encoding for the input or output domain, is generally blunt ligated into the open plasmid generating the random domain insertion library. In addition, circular permutation of the insert gene can be used to create another layer of diversity, thus increasing the overall diversity of the protein switch library [11, 46].

## 3. Periplasmic Binding Proteins as Input Domain Provide Immense Ligand-Binding Diversity

Periplasmic binding proteins are a superfamily of bacterial receptors that mediate chemotaxis and solute uptake [47, 48]. PBPs are fundamental components of ATP-binding cassette (ABC) transport systems and are located in the periplasm of Gram-negative bacteria. In Gram-positive bacteria and archaea, the periplasmic binding component is replaced by a membrane bound lipoprotein, which acts in a similar manner. PBPs have been identified for a wide variety of ligands including amino acids, dipeptides, oligopeptides, carbohydrates, lipids, peptidoglycans, vitamins, drugs, metabolic products, quorum sensing molecules, hormones, metal ions, and anions [49, 50]. PBP-structure has also been identified in domains of DNA repressors such as LacI, eukaryotic receptors such as GluR2, and enzymes [47, 48].

**Figure 3:**
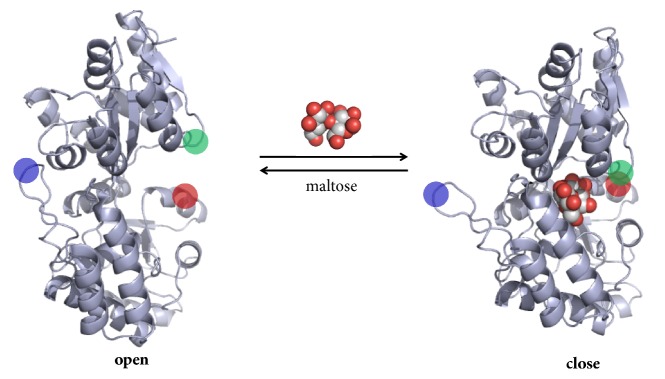
Structural representation of the conformational changes of PBPs. The figure shows a periplasmic maltose-binding protein (MBP) from* E. coli* in its open apo-form (PDB accession 1OMP) and in its closed holo-form upon maltose binding (PDB accession 1ANF). The flexibility of the hinge region allows the large conformation change in MBP. Red and green circles indicate regions which are separated in the open form and are proximate in the closed form. The blue circle shows a region in which packing is changed between open and close forms. Structural representation was rendered in PyMol.

Members of the PBPs superfamily show high sequence diversity, but protein structure is usually conserved [49]. They consist of two domains linked by a flexible hinge region being the binding site located at the interface between the two domains. PBPs may adopt two different conformations (Figure 3): an open conformation when no ligand is bound (apo-form) and a closed conformation upon binding (holo-form). These conformations are interconvertible by a relatively large bending movement around the hinge region [51]. Two different structural classes have been described, classes I and II, which differ in their topology [49]. Currently, there are 302 PBP structures deposited in the Protein Data Bank (research made with the term “periplasmic binding protein”).

Bioengineers have been using the intrinsic properties of PBPs to build biosensors and other elements allosterically modulated [49]. Among these properties, it is possible to highlight (i) high ligand affinity (range from nM to *μ*M), solubility, stability [52], and (ii) the large ligand-mediated conformational changes [53], which can allow molecular communication between linked domains through conformational coupling. Although conformational changes in proteins due to ligand binding are ubiquitous and integral in biological systems, it is often challenging to link ligand-induced conformational changes to a resulting biological function.

An evident limitation to engineer novel biosensors is finding a specific PBP carrying all required properties for the tailored sensor. While still difficult to build new PBPs through* de novo* protein design, three distinct approaches can be applied for overcoming this issue: (i) mining novel natural PBPs using genome or metagenome strategies [70, 71]; (ii) using computational design methods upon known protein scaffolds to create new protein–ligand interfaces [72]; and (iii) the combination of computational protein design and directed evolution to generate affinity for other ligands in a specific PBP [56, 73]. Several PBPs have been created using these approaches expanding the possibilities to design novel biosensors.

## 4. Protein Switches Based on Antibiotic Resistance

**Figure 4:**
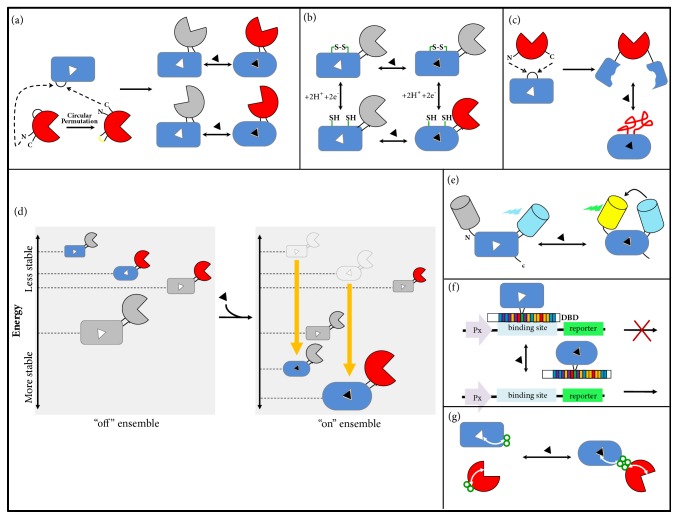
Domain insertion strategies for converting PBPs into switchable proteins. (a) In a typical domain insertion, the inserted domain (red) has proximal N- and C-termini. These natural termini can be closed by a linker and new terminals can be created by circular permutation. The new termini of the circular permuted protein are inserted into a surface loop of the acceptor protein, PBP (blue). Combination of circular permutation and domain insertion increases the overall diversity of the protein switch library, generating different geometries. (b) Multi-input protein switches can be created by introducing disulfide bonds (green) to keep the PBP domain in an unbound (closed) conformation, which keeps the output domain in an “off” state. This example shows an AND gate logic in which the presence of both inputs, reduction of the disulfide bonds and ligand, is necessary to activate the output domain. (c) Mutually exclusive folding. The terminals of the inserted domain are far away from each other. This configuration creates a structural tug-of-war between the domains. Ligand binding stabilizes the PBP fold, which mechanically unfolds the output domain. (d) Ensemble model of allostery. In an “off” ensemble, the most probable chimera state has inactive input and output domains. Ligand binding remodels the population in the ensemble by increasing the stability of those states that bind the ligand and the most probable state has active domains. (e) FRET-based biosensors. FRET depends on the physical distance between a donor and an acceptor fluorophore. The PBP conformational change in response to ligand approximates the fluorescent proteins allowing energy transfer. (f) Inducible transcription factors can be designed through fusion between PBPs and DNA-binding domains (DBDs), leading to versions of DBDs that respond to new ligands. (g) Rational domain insertion can be possible through computational analysis (statistical coupling analysis-SCA). The analysis of the network of coevolving residues can predict distant sites on the surface. These sites can be used for coupling fusions. PBPs are shown as a rectangular (closed form, unbound) or an oval (open form, bound) shape. A grey color of the domain indicates that the protein is inactive. The signal that modulates the switch is showed as a black triangle.

A cornerstone of protein engineering is the development of high throughput screening/selection systems (HTS). Construction of such systems has been used not only to generate improved proteins but also to design whole-cell biosensors that translate cellular signals into quantifiable outputs [74]. One such example is the use of antibiotic resistance as output signal in which the modulation of enzymatic activity results in the alteration of cellular survival capacity in the presence of the antibiotic. This allows the screening of large libraries (e.g., > 10^7^ members), performing even better than contemporary automated platforms. Therefore, a protein switch carrying an enzyme that provides resistance to antibiotic in its output domain is an attractive biosensor for both* in vivo* (e.g., whole-cell biosensors for HTS) and* in vitro* (e.g., colorimetric enzymatic assay) applications. Ostermeier and coworkers have developed a family of growth/no-growth biosensors for maltose using the TEM1 b-lactamase (BLA) enzyme as output domain. Initially, it was demonstrated that BLA could be randomly inserted into the maltose-binding protein (MBP) from* E. coli* to generate switch proteins. After HTS steps, a chimeric protein was selected in which BLA activity was positively modulated by maltodextrins, either* in vivo* or* in vitro*. However, this selected switch showed low dynamic modulation (≤ 1.8-fold) [54]. Next, an improved MBP-BLA was developed by random circular permutation (cp) of the BLA gene followed by random insertion of the cpBLA library into the MBP gene (Figure 4(a)) [55]. This approach generated a switch protein (termed RG13) with BLA catalytic efficiency increased 25-fold in the presence of maltose. BLA activity in RG13 also gained responsiveness to a second input, the Zn^2+^ion, which worked as a negative modulator, showing that novel properties can emerge from chimerogenesis [75]. Afterwards, an iterative approach involving alternating random domain insertion and random circular permutation generated a switch with 600-fold increase in BLA activity upon maltose binding [56]. This protein switch, termed MBP317–347, has a cpBLA (BLA170) inserted into a loop of MBP containing a key active site amino acid. Furthermore, mutations were introduced into the ligand-binding site of the MPB domain of the MBP317–347, conferring the ability to bind sucrose without eliminating maltose binding [56]. In addition, MBP317–347 also showed low affinity for glucose and the potential to be used as a biosensor of metabolic biomarkers from tumors [76].

In order to explore the structural basis of local amino acid residues important to the allosterism generated by domain insertion, Choi and coworkers [61] used the engineered protein MBP317-347 for the construction of 285 mutants. MBP317-347 binding pocket was the target for all possible amino acid replacements at 15 positions, which were selected due to their known involvement with maltose binding. Interestingly, the allosteric function and the “phenotype switch” were resistant to mutations, but a few of them affected the affinity of MBP317-347 for maltose and also the switching behavior. Mutations in positions E153 and R66 caused a decrease in BLA activity in the absence of maltose, enhancing the switch effect in the presence of the effector. Results obtained in this study suggest that the design of improved switches could be achieved by focusing on the effector-binding site to generate mutations which could enhance the modulating effect.

In addition, switches sensing ribose, glucose, and xylose have also been created through fusion of BLA into ribose binding protein (RBP) (≤7-fold), glucose binding protein (GBP) (≤2-fold), and xylose binding protein (XBP) (≤4-fold) from* E. coli*, respectively [33]. Although these switches share paralogous PBPs as input domain, and some overlap in sites for switch insertion, successful switches at these sites required different circular permutations of the BLA and different linkers between the domains.

### 4.1. Multi-Input Protein Switches

In seeking to make biology “programmable,” synthetic biology approaches have been applied to engineer cells that respond to molecular signals with a tailored behavior. Many of these approaches focus on the creation of multi-input logic gates to improve the cell decision-making efficiency. Proteins have the potential to perform all 16 possible two-input logic gate operations [77]. In addition, they have proven to be an important alternative to classical transcriptional networks for logic gates design, offering the advantages of simplicity, speed, and reduced consumption of cellular resources [25, 58].

The chimeric MBP-BLA protein RG13 showed an allosteric mechanism dependent on conformational changes in the MBP domain [55, 75]. As a consequence of RG13 chimerogenesis, the BLA domain gained a novel noncompetitive input (Zn^2+^), and the switch effect was modulated by two-input signals [75]. In a recent work, RG13 was rationally redesigned to demonstrate the potential for a single protein to show a multi-input control and different logic gates behavior [58]. In this study a redox-control was integrated in RG13 through introducing disulfide bonds to keep the MBP domain in either an open or closed conformation (Figure 4(b)). In the closed conformation BLA activity is “on” and it is switched to “off” state when MBP changes to an open conformation. Thus, besides the maltose-regulated allostery of RG13, the switch also became dependent on the disulfide bond, which is controlled by redox agents. These RG13 variants were also applied to create AND, ORN, and YES logic gates behavior, offering a protein-based alternative to produce a functional output from multiple inputs [58]. Moreover, since an electrochemical signal can also be used to reduce disulfide bonds, the engineered RG13 variants showed a voltage-dependent switch effect [59]. These studies suggest that disulfide containing protein switches are a useful platform to create multi-input switches with synthetic biology applications, redox-controlled biosensors, and also bioelectronic sensors.

### 4.2. Protein Switches Based on an Ensemble Model of Allostery

In the last decades, the classic view of allostery has expanded, and the ensemble model emerged. This model statistically describes the existence of dynamic conformational ensembles rather than considering proteins allosterically regulated as a static two-state model (active and less active). [78–80]. A protein switch can show a behavior compatible to ensemble allosteric model, in which the remodeling of the landscape energy is responsible for tuning the protein activity (Figure 4(d)). In order to design switchable enzymes based on an ensemble allosteric model, a nonallosteric enzyme was converted into an allosteric protein through modulation of the conformational entropy [60]. Based on the previously identified nonallosteric bifunctional chimera (termed c4 [81]) where cpBLA was inserted into MBP, the linker region was engineered to increase the conformational flexibility of the chimera in the absence of maltose (effector). These engineered linkers created intrinsically disordered regions that reduced enzyme activity in the absence of the effector. The addition of maltose stabilized active states of the enzyme, specifically at higher pH or temperature, generating a switch effect. Thus, the modulation of the conformational entropy allowed converting a nonallosteric protein into a multi-input switch which requires a particular environmental condition (temperature or pH) and the presence of the effector for its activation [60].

## 5. Protein Switches Based on Fluorescence

Fluorescent protein biosensors (FPBs) are probes containing a sensing domain that recognizes a target molecule and a reporter module that generates the fluorescence signal [82]. These biosensors permit quantifying analytes within and between cells, both* in vivo* and* in vitro,* through changes in fluorescence properties (e.g., excitation or emission wavelengths, intensity, and lifetime of the excited state). In addition, FPBs allow the measurement of temporal and spatial dynamics of targeted cellular events in real time [82].

Single FPBs (SFPBs) are fluorescent sensors encoded by a single polypeptide chain composed of a circularly permuted fluorescent protein (cpFP) inserted into a specific ligand-binding domain [82]. Marvin and coworkers [63, 64, 66] developed SFPBs with high signal-to-noise ratio by the insertion of cpFPs into PBPs. In order to generate biosensors for disaccharides, organophosphorus compounds, and glutamate, cpGFP was inserted into* E. coli* maltose-binding protein MalE [64], phosphonate-binding protein PhnD [63], and glutamate-binding protein GltI [66], respectively. Residues predicted to undergo dramatic rearrangement upon ligand binding were chosen for the cpGFP insertion, and the linkers between cpGFP and PBP were optimized. Binding of ligand induces the PBP conformational changes switching the biosensor to a bright state. The GltI/cpGFP chimera, termed iGluSnFR, showed specific response for* in situ* glutamate signal in neurological systems from worms, zebrafish, and mice [66].

In a recent study, Wu and coworkers [67] also developed a high-affinity biosensor for glutamate termed R-iGluSnFR0.1. This protein was created replacing the cpGFP domain of iGluSnFR by a circularly permuted red FP (cpRFP). Then, the switch effect was improved through linker optimization and iterative rounds of directed evolution. In addition, a second variant was constructed by circular permutation of GltI instead of RFP. The cpGltI was inserted into nonpermuted RFP at the same position as previously identified for iGluSnFR, leading also to a high-affinity biosensor for glutamate (R^ncp^-iGluSnFR1). Both variants, R-iGluSnFR0.1 and R^ncp^-iGluSnFR1, worked successfully as biosensors for glutamate when targeted to the surface of HEK-293 cells [67]. These results show that the changing of protein topology may offer a new diversity layer for engineering fluorescent biosensors.

In an elegant approach, Nadler and coworkers [62] describe a general approach, termed domain insertion profiling with DNA sequencing (DIP-seq), to identify functional metabolite responsive SFPBs. As proof of concept to validate the methodology, they created domain insertion libraries between* E. coli* MBP and cpGFP, since the SFPB composed by MBP and cpGFP is a benchmark to generate allosteric fusions [54, 64]. Using* in vitro* transposition, MBP tolerance to random cpGFP insertion was evaluated by functional screening of the library with fluorescence-activated cell sorting (FACS) in the presence of maltose. Deep sequencing analysis showed that ~57% of MBP residues contained productive insertions of cpGFP. With this approach, maltose biosensors with a greater switching dynamic than the one previously published by Marvin and coworkers [64] were found. Moreover, with the aim of developing novel biosensor for trehalose, cpGFP was inserted into the D-trehalose/D-maltose-binding protein (TMBP) from* Thermococcus litoralis* [83] by* in vitro* transposition. This domain insertion library was enriched with switch proteins through iterative rounds of FACS screening in the presence or absence of trehalose. The best selected variant was then optimized by varying the linkers length (0-3 amino acid) connecting cpGFP and TMBP generating the variant Tre-C04. This switch works as a high-affinity and high-selectivity biosensor of trehalose showing ~6-fold fluorescence induction in the presence of the sugar. Deep sequencing of domain insertion libraries allowed mapping allosteric hotspots and functional FPBs for both MBP and TMBP [62].

Fluorescence resonance energy transfer (FRET) is a process of energy transfer between two fluorophores, and this mechanism is dependent on the physical distance between the donor and the acceptor. This technique can be used to measure the proximity at molecular distances (Å) and therefore phenomena that cause molecular proximity/distancing [84]. Hence, FRET is an interesting tool for the study of protein switches that involve physical molecular proximity. Domain insertion also has been used to develop FRET-based biosensors (Figure 4(e)). Glucose and glutamate FRET-based biosensors were designed through insertion of the enhanced cyan fluorescent protein (ECFP) into* E. coli* glucose/galactose-binding protein (MglB) and glutamate/aspartate-binding protein (YbeJ), respectively [17]. A yellow fluorescent protein (YFP) was attached either at the N- or C-terminal, producing chimeric proteins with a high-FRET ratio upon ligand binding, suggesting an indirect allosteric regulation during the hinge-bending motion [17].

Bogner and Ludewig [65] used a similar strategy to develop an arginine biosensor through the insertion of citrine, a YFP, into a glutamine binding protein (QBP) bound to an enhanced ECFP. This biosensor was tested in* E. coli* and in* Arabidopsis*, in which exposure to arginine and ornithine increased the fluorescence ratio, suggesting this sensor could be used as a tool for more comprehensive studies of* in vivo* dynamics in plants [65]. Another work using FRET strategy aimed at developing a sugar biosensor that could be used for physiological analyses to select small interfering RNAs (siRNAs) related to the regulation of sugar flux [85].

## 6. Protein Switches Based on Allosteric Transcriptional Regulators

Recognition of an external stimulus (input) resulting in a particular cellular response (output) is an efficient approach to developing engineered cells with biosensor functions [86]. In most organisms, the ability to respond to an input is often closely linked to the fine control of gene expression. A key strategy for optimizing this control is the design and rearrangement of regulatory elements for tuning gene expression. Although many tools have been developed, most are focused on elements that act in* cis* (e.g., promoters, ribosome binding sites, and terminators) and are limited by compatibility, ease of implementation, difficulty to optimize gene expression under multiple conditions, and undesirable effects in the native physiology of the microorganism [87]. Many of these problems could be overcome with the development of* trans*-acting DNA-binding transcription factors (TFs) that could control the expression of a target gene without changing its native regulation.

Naturally occurring inducible TFs have been broadly used as important elements to control gene expression in response to a target molecule. However, these natural proteins have limited recognition spectrum. One approach to engineer new conditional TFs is to use directed evolution or rational design to fuse, by domain insertion, PBPs with DNA-binding domains (DBDs), leading to versions of DBDs that respond to new ligands (Figure 4(f)).

An attractive DBD family comprises the zinc finger proteins (ZFPs), especially Cys2-His2 class, since each “finger” of the ZFP binds to a distinct 3 bp DNA sequence, and multiple “fingers” can be fused in tandem to engineer new ZFPs [88]. In order to expand the toolkit of inducible TFs, Younger and coworkers [68] inserted a ZFP (BCR-ABL1), into MBP. BCR-ABL1 binds to its cognate 9 bp DNA-binding site with three tandem fingers [89] and is optimized to work as a transcriptional repressor in* E. coli* by sterically blocking RNA polymerase binding to the promoter. For this purpose, BCR-ABL1 was inserted after residue 316R into MBP, a position previously identified to generate an MBP/BLA switch protein [55]. The insertion generated an MBP/BCR-ABL1 inducible repressor being the expression of its target gene activated by the presence of maltose with moderate dynamic modulation (≤ 4-fold induction). Even though the MBP/BCR-ABL1 mechanism of action is not clear, the authors hypothesized that maltose binding to the MBP domain changes the ZFP domain conformation to a state not capable of binding to DNA [68]. In a different approach, directed evolution was used to randomly insert BCR-ABL1 into MBP using* in vitro* transposition [69]. After four rounds of screening with FACS, in the absence or presence of maltose, three unique maltose-responsive biosensors were identified (MBP insertion points: 270A, 277A, and 335P). However, even after linker optimizations all the variants showed a moderate dynamic modulation (≤ 4-fold induction) [69].

## 7. Protein Switches Based on Phenotype

As mentioned before, when developing protein switches by random domain insertion, the identification of responsive chimeras relies on an efficient screening method. When this screening is made based on a cellular phenotype, the switching behavior selected could be due to mechanisms other than allostery, for instance, by accumulation of the active state into the cell. In 2011, Ostermeier's group published a study in which a library of cpBLA was inserted in residue 317 of MBP [81]. The library was screened for chimeras that confer antibiotic resistance to* E. coli* in the presence of maltose. Among the 34 colonies selected, only 4 of them presented an allosteric behavior. The other colonies were later called “phenotypic switches”. In this example, a higher cellular accumulation of the fused protein was detected in the presence of the inducer, indicating that this phenotype was a result of the fused protein interaction with maltose [81]. Then, they sought to investigate the mechanism of these phenotypic switches [90] using two previously constructed chimeric variants [81], in which BLA was inserted after residue 316 of MBP with a linker (c4) or without a linker (Ph7) between BLA and residue 319 of MBP. Ph7 behaved as a phenotypic switch and c4 showed no switching behavior, suggesting that the linker might be the cause of the different performance. Therefore, they tested a set of chimeric proteins varying the linker length between BLA and MBP. Initially, two* E. coli* strains were transformed with the variants and there was a significant difference of the phenotypic switch effect between the strains, suggesting that the behavior was strain dependent and that cellular factors might play an important role. The authors proved that Ph7 was more susceptible than c4 to proteolysis and that in the presence of maltose both proteins were less hydrolyzed [90]. Therefore, they suggest that maltose binding to MBP partially protects proteins from being digested. Another reason for protein accumulation in the cell could be a disturbance on thermodynamic stability. The authors suggest that the longer the linker, the higher the thermodynamic stabilization in the presence of maltose. Thus, maltose binding to MBP in the fused protein seems to be responsible for increasing protein accumulation due to lower protease susceptibility and a higher thermodynamic stability.

## 8. Mutually Exclusive Folding

Differently from the previous examples in which the inserted protein has its N- and C-termini close, in mutually exclusive strategy the terminals are distant from each other. This configuration forbids both domains from being simultaneously folded. Thus, the free energy of one domain is used to mechanically unfold the other, creating a structural tug-of-war between the domains (Figure 4(c)) [91–94]. Linkers can be introduced between domains; however, they cannot be too long to cause uncoupling of the structure, neither too short to prevent intramolecular folding [92]. Loh's group [95, 96] applied mutually exclusive folding principles to develop protein switches induced by a mechanism known as domain swapping. Domain swapping is a structural phenomenon by which identical proteins exchange segments in reciprocal fashion to yield oligomerization. Ubiquitin (Ub) or FK506 binding protein (FKBP) was inserted into functionally inactivated point mutants of ribose binding protein (RBP) from* Thermoanaerobacter tengcongensis*. Ub and FKBP worked as ‘‘lever” proteins that split RBP forcing it to first unfold then refold via swapped dimer. The mutant RBP alone cannot bind ribose; only by swapping and dimerization its function is restored. The drug FK506 worked as input signal to trigger swapping activation, and RBP worked here as an output domain [95, 96].

## 9. Future Perspectives

The ability of coupling an input event to an output signal in a protein switch has tremendous utility for the development of biosensors and also regulation of biological systems. The approaches outlined here show how the power of domain insertion can be harnessed for fine-tuning a protein activity and to evolve allostery. However, the scourge for designing switchable proteins by domain insertion is its low-throughput nature due to the difficulties in predicting the right insertion sites for domain coupling. Therefore, through many successful examples described above it was necessary to try many different fusions, followed by extra steps of circular permutation, linker optimization, using tedious and time-consuming screenings. Thus, overcoming this limitation is paramount to expanding protein switches applications. Engineering allosteric switches through rational insertion would be an ideal approach for biosensor designing. Ranganathan's group has used a statistical coupling analysis (SCA) to identify physically connected networks of coevolving amino acids which link the functional site of a protein with a distant surface site [97, 98]. Reynolds and coworkers [98] have used this computational tool for scanning domain insertion positions in the enzyme dihydrofolate reductase (DHFR) and inserted a light-sensitive domain (LOV2) into these “hotspots” positions, which allowed the identification of a chimeric protein regulated* in vivo* by light. Thus, linking of existing allosteric networks in the individual protein domains could lead to allosteric connections [99] (Figure 4(g)). The large data set generated by previous work on building protein switches could serve to expand this approach.

In addition, recent studies have explored deep sequencing analysis to address how domain insertion affects allostery [27, 62]. These datasets can help us understand better the fitness landscape for protein switches and increase the success rate for domain coupling.

PBPs conformational change has shown a powerful feature for allosteric coupling in protein switches. Nonetheless, the creation of a novel biosensor depends on the availability of a PBP to bind to the target ligand, undergo conformational changes, and be able to either accept domain insertions or have an appropriate distance between the N- and C-termini to be inserted. Thus, the limited repertoire of natural PBPs available to date is a barrier for the development of switches. Several approaches can be applied to expand the number of PBPs available, such as metagenomic [70, 71], directed evolution [56, 73], and computational design strategies [72].

In the future, hence, with continuous computational advances, it may be possible to design allosterically regulated biosensors by engineering novel PBPs, and predicting insertion sites for perfect domain coupling. This suggests that there is still plenty of scope for the engineering of switch proteins. One example is the small number of engineered TFs. Combining PBPs with DNA-binding proteins (e.g., TALE or CRISPR/Cas9) to design novel inducible TFs would have extraordinary importance. In addition, many of the studies described here are specific to bacteria. The adaptability of these tools to other platforms such as mammalian cells, fungus, and plant cells would greatly increase its biotechnological impact.

Given the rapid development of switches engineered by domain insertion, it is likely that in the future a wide range of tailored biosensors, working* in vivo* or* in vitro*, will be available for a wide variety of applications.

## Figures and Tables

**Table 1:** Selected examples of PBP-based protein switches generated by domain insertion*∗*.

**Function modulated *in vivo***	**Input domain (PBPs)** ^**a**^	**Output domain** ^**b**^	**Method of creating switch**	**Input signal**	**Kd (** ***μ*** **M)**	**Variant name: switch effect**	**Ref**
	MBP	BLA	Random insertion of BLA into MBP	Maltose	3.2	T164-165: 1.6-fold^c^	[54]
					1.7	T164-165-H: 1.8-fold^c^	
	MBP	BLA	Circular permutation of BLA and random insertion into MBP	Maltose	5.5	RG13: 25-fold^c^	[55]
	MBP	BLA	Iterative circular permutation and random insertion of BLA into MBP	Maltose	0.5	MBP317–347: 600-fold^c^	[56]
			Five residues in the maltose binding site were randomized generating a switch variant that binds to sucrose	Maltose	23	MBP317–347/5-7: 86-fold^c^	
				Sucrose	0.7	MBP317–347/5-7: 82-fold^c^	
	GBP	BLA	cpBLA was randomly inserted into the PBP	Glucose	ND	MRD2col9: 2-fold^c^	[33]
	RBP			Ribose	ND	P1. F10: 7.2 fold^c^	
	XBP		Random insertion of BLA into XBP and linker optimization	Xylose	ND	XBPBLA12: 4.4-fold^c^	
**Antibiotic resistance**	MBP	BLA	Site-directed mutagenesis of I329 residue of RG13	Maltose	0.67	I329A: 23-fold^c^	[57]
					0.63	I329K: 32-fold^c^	
					25.6	I329P: 12-fold^c^	
					0.55	I329W: 20-fold^c^	
	MBP	BLA	Disulfide bonds were rationally introduced in RG13	Maltose	ND	RG13-AND2: 2-fold^d^	[58]
					ND	RG13-ORN2: 8-fold^d^	
					ND	RG13-YES: 2-fold^d^	
				Maltose (+GSH)	ND	RG13-AND2: 8-fold^d^	
					ND	RG13-ORN2: 16-fold^d^	
					ND	RG13-YES: 4-fold^d^	
	MBP	BLA		Maltose	ND	RG13-AND2: 1.38^c^	[59]
				Maltose (+ e^−^)	ND	RG13-AND2: 3.59^c^	
	MBP	BLA	Linker modification of nonallosteric MBP-BLA (c4) to search for emergence of allostery through modulation of the conformational entropy	Maltose (heat or OH^−^)	ND	c4-4G: 32-fold^d^	[60]
	MBP	BLA	Point mutations at selected residues of the maltose binding pocket of MBP317-347	Maltose	499	E153D: 2,360-fold^c^	[61]

	MBP	GFP	Circular permutation of GFP and random insertion into MBP	Maltose	2.8	Mal-B2: 8.1-fold^d^	[62]
	TMBP			Trehalose	0.053	Tre-C04: 6.3-fold^d^	
	PhnD	GFP	Circular permutation of GFP and insertion at four positions of PnBP based on structure. Mutagenesis of the inter-domain linkers	2AEP	37	EcPhnD90- cpGFP.L1ADΔΔ. L297R,L301R: 1.5-fold^d^	[63]
	MBP	GFP	cpGFP was inserted in selected places of MBP. Further linker optimization	Maltose	4.5	MBP165-cpGFP: 0.2-fold^d^	[64]
					3	MBP165-cpGFP.PPYF: 2.5-fold^d^	
					1.3	MBP175-cpGFP.L1-HL: 0.5-fold^d^	
**Fluorescence**	MglB	YFP	CFP (FRET donor) insertion into selected sites of MglB,with YFP at either C-term or N-term of MglB	Glucose	600	FLII^12^Pglu-600*μ*: 2.66-fold^c^	[17]
	GltI	YFP	CFP (FRET donor) insertion into selected sites of GltI,with YFP at C-term of GltI	Glutamate	1	FLII81PE-1*μ*: 3.8-fold^c^	
	QBP	CFP	QBP coding sequence was amplified as two fragments. One was inserted at the linker region of YFP-CFP cassette and the other at the N-term yielding QBP-YFP-QBP-CFP	Arginine	2100	QBP/Citrine/CFP: 1.3-fold^c^	[65]
				Ornithine	2000	QBP/Citrine/ECFP:1.1-fold^c^	
	GltI	GFP	cpGFP was inserted in a selected site of GltI. Further linker optimization	Glutamate	107	GltI253.L1LV/L2NP: 4.5-fold^c^	[66]
				Aspartate	145	GltI253.L1LV/L2NP: 2-fold^c^	
	GltI	RFP	Nonpermuted RFP was inserted in a selected site of cpGltI. Further linker optimization and directed evolution	Glutamate	0.9	R^ncp^-iGluSnFR1 4.8-fold^c^	[67]

**Enzymatic activity**	XBP	XynA	XynA was inserted into selected places of XBP. Further linker variation	Xylose	0.16	2621B: 1.49-fold^c^	[22]
	XBP	XynA	XynA was randomly inserted in XBP	Xylose	ND	XynA–XBP271: 1.5-fold^c^	[23]

**Control of gene expression**	MBP	ZFP	The zinc finger protein BCR-ABL1 was inserted into MBP	Maltose	ND	SP: 3-fold	[68]
	MBP	ZFP	BCR-ABL1 was randomly inserted into MBP. Further linker optimization	Maltose	ND	316R: 4-fold^d^	[69]
						277A: 1.2-fold^d^	
						270A: 2-fold^d^	
						335P: 2.4-fold^d^	

^**∗**^Studies in which many variants were constructed, and only those displaying the best switch effect were considered.

^a^MBP, maltose binding protein; GBP, glucose binding protein; RBP, ribose binding protein; XBP, xylose binding protein; TMBP, trehalose/maltose-binding protein; PhnD, phosphonate-binding protein; MglB, glucose/galactose-binding protein; QBP, glutamine binding protein; GltI, glutamate-binding protein.

^b^BLA, TEM1 *β*-lactamase; GFP, green fluorescent protein; YFP, yellow fluorescent protein; CFP, cyan fluorescent protein; XynA, xylanase from *Bacillus subtilis*; ZFP, zinc finger protein.

^c^*in vitro* or  ^d^*in vivo* assay values in the presence of the effector divided by the value in the absence of the effector.
